# Enabling next generation systems biology: a conversation with M. Madan Babu

**DOI:** 10.15252/msb.199376

**Published:** 2019-12-05

**Authors:** Thomas Lemberger, M Madan Babu

**Affiliations:** ^1^ EMBO Heidelberg Germany; ^2^ MRC Laboratory of Molecular Biology Cambridge UK

**Keywords:** S&S: Ethics

## Abstract

*Molecular Systems Biology* warmly welcomes its new academic Chief Editor, M. Madan Babu. Madan shared his thoughts on the evolution of the field and the importance of bridging disciplines to enable next generation systems biology.

## Graphical abstract

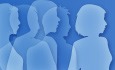

Since its launch in 2005, *Molecular Systems Biology* has developed strong ties with the scientific community through its Senior Editors and academic Advisory Editorial Board as well as by the continuous interactions of the editorial team with scientists in the field. Strengthening the journal's roots in the systems biology community has always been our priority, and we are therefore delighted to welcome M. Madan Babu as the new academic Chief Editor of *Molecular Systems Biology*.

Technological and conceptual innovations in systems biology have transformed the way biological systems are investigated. Our journal has followed the evolution of systems biology since it early days, and the diversification of the field has prompted us to maintain a broad and inclusive scope. *Molecular Systems Biology* offers a home to a broad range of studies across the many flavors of systems biology and synthetic biology. Our new Chief Editor will engage with the community to cultivate this open‐minded tradition, acting as an ambassador for the journal and helping bridge fields and disciplines.

At the editorial level, the *Molecular Systems Biology* editorial team will continue running the transparent peer‐review process and applying the policies shared by the EMBO Press family of journals.

To get our readers to know Madan better, we asked him a few questions about his new role and his vision for the journal and the field.

## Q. Why did you accept the role of Chief Editor of *Molecular Systems Biology*?

Systems Biology as a field is undergoing enormous transformations in terms of the diversity of questions that are being investigated. *Molecular Systems Biology* has been at the forefront in establishing the field. I see the role of Chief Editor of the journal as a unique opportunity to identify and bring together scientific ideas and concepts that not only push the boundaries but also cut across these emerging and developing sub‐disciplines. It will be a privilege to help catalyze, transform, and shape the evolving landscape of systems biology, systems medicine, and synthetic biology research.

## Q. Will the scope of *Molecular Systems Biology* change?

The scope of *Molecular Systems Biology* will certainly expand with one guiding principle: breaking the silos and boundaries between traditional disciplines and being more integrative, quantitative, and multi‐scale in the nature of the scientific questions addressed. As new technologies and initiatives that probe living systems at all scales continue to advance, we hope that the journal will serve as the home for new sub‐disciplines as well as previously well‐established areas of systems biology and synthetic biology.

Our goal is to cover systems and synthetic biology research across the disciplines such as molecular biology, genetics and genomics, physiology and medicine, chemical biology, biological physics, mathematical biology, network biology, evolutionary biology, biotechnology, pharmacology, neurobiology, immunology, as well as cell and developmental biology at all scales and resolution: from studies of systems at the atomic level (e.g., populations of molecules) to network of interactions between molecules and cells (e.g., biomolecular and neural circuits) to those pertaining to the level of the entire population of a species and ecosystem (e.g., genome variation and microbiomes).

## Q. How did the field evolve since *Molecular Systems Biology* was launched, back in 2005?

Having followed the development of the journal since its inception in 2005, it is striking to see the field evolve in its ability to study living organisms in the following aspects: the scale of investigation (e.g., from single molecules to entire microbial communities), the level of resolution (e.g., from bulk approaches to single cell measurements), and the degree of accuracy (from estimates to precise quantification of the abundance of biomolecules).

There have also been major breakthroughs in quantifying attributes of biological molecules as well as in probing mechanistic details of biological processes. In the coming years, I believe scientists will push these limits and become more integrative and quantitative in bridging these scales. I am personally excited to witness these developments and the next frontier of research in systems biology in the coming years.

## Q. How do you view the interaction between systems biology and synthetic biology?

In my view, systems biology and synthetic biology go hand in hand. There has been enormous progress in unraveling the complexities of naturally occurring biological systems especially with advances in genomics, transcriptomics, proteomics, and metabolomics. Such systems biology approaches have not only enabled fundamental discoveries but also elucidated general principles. The future of systems biology will involve actively bridging the sub‐disciplines by linking the multiple networks of biological entities to gain a deeper understanding of the emergence of complexity. Among the many possibilities, such advances will allow us to uncover novel biological mechanisms, predict the impact of perturbation at the mechanistic level, and identify entities to target in an organism to achieve the desired phenotypes.

At the same time, we have seen how synthetic biology has evolved from building and characterizing the properties of a few interacting molecules to being able to robustly generate predictable phenotypes of fairly large networks of molecules governed by several genes and microbial communities. The next frontier of synthetic biology will involve engineering entire genomes, starting with microbes, to create synthetic organisms and ecosystems that are capable of performing novel functions and exhibiting specific phenotypes in different environments.

Such advances will involve development of new experimental methods, computational approaches, and theoretical and conceptual frameworks involving multi‐scale modeling and data integration. Developments in these directions hold enormous promise for human health, environment, and society, and we look forward to publishing such studies in *Molecular Systems Biology*.

## Q. What are the current major challenges in the biomedical sciences?

One of the major challenges in biomedical sciences is to interpret the genome of humans (and pathogens), as well as understand how variation in their genomes can lead to healthy phenotypic diversity or disease. Solving this problem will allow us to assess how the environment interacts with the genome to determine phenotypes.

Developments in these areas will not only provide mechanistic insights but also help appreciate the role of nature and nurture in various health related issues. This will have significant impact in developing treatment strategies, therapeutic interventions, and recommendations to life‐style changes that can improve human health.

## Q. What would be your advice to young scientists who hesitate between a career in fundamental research versus translational or applied sciences?

This is a very important consideration for the next generation of scientists. I would advise young scientists not to be strictly binary in their choice or box themselves into one or the other. Of course, it is important to focus and follow one's passion but it is equally important to keep track of developments beyond the chosen career path. This would ensure that any basic science findings can be effectively translated into a specific application (or have translational impact) and vice versa (i.e., findings from translational studies providing fundamental insights into a basic biological process) when opportunities present themselves.

## Q. Do you think that the time has come for translating systems approaches to global health and environmental issues?

I strongly believe so. There are already a number of public and private initiatives that are aiming to apply systems approaches to global health (e.g., the WHO One Health Initiative and several systems medicine and precision medicine efforts) and environmental issues (e.g., TARA Oceans Project and the Earth BioGenome Project). Systems approaches that bridge scales and integrate diverse data types will allow effective implementation of these efforts. These efforts and other future initiatives will have a significant impact on both global health and the environment, including affordable and clean energy initiatives and climate action.

